# A Simple UPLC/MS-MS Method for Simultaneous Determination of Lenvatinib and Telmisartan in Rat Plasma, and Its Application to Pharmacokinetic Drug-Drug Interaction Study

**DOI:** 10.3390/molecules27041291

**Published:** 2022-02-15

**Authors:** Yanjun Cui, Ying Li, Xiao Li, Liju Fan, Xueru He, Yuhao Fu, Zhanjun Dong

**Affiliations:** 1 National Clinical Drug Monitoring Center, Department of Pharmacy, Hebei Province General Center, Shijiazhuang 050051, China; 15227160198@163.com (Y.C.); lyyaoda@126.com (Y.L.); lx15132106723@163.com (X.L.); lijufan91@163.com (L.F.); hxr9115@163.com (X.H.); ya202202@126.com (Y.F.); 2 Department of Pharmacy, Hebei General Hospital, Shijiazhuang 050051, China

**Keywords:** UPLC-MS/MS, lenvatinib, telmisartan, pharmacokinetics, drug-drug interaction

## Abstract

Lenvatinib is a multi-targeted tyrosine kinase inhibitor that inhibits tumor angiogenesis, but hypertension is the most common adverse reaction. Telmisartan is an angiotensin receptor blocker used to treat hypertension. In this study, a simple ultra-performance liquid chromatography-tandem mass spectrometry method was developed for the simultaneous determination of lenvatinib and telmisartan, and it was applied to the pharmacokinetic drug interaction study. Plasma samples were treated with acetonitrile to precipitate protein. Water (containing 5 mM of ammonium acetate and 0.1% formic acid) and acetonitrile (0.1% formic acid) were used as the mobile phases to separate the analytes with gradient elution using a column XSelect HSS T3 (2.1 mm × 100 mm, 2.5 μm). Multiple reaction monitoring in the positive ion mode was used for quantification. The method was validated and the precision, accuracy, matrix effect, recovery, and stability of this method were reasonable. The determination of analytes was not interfered with by other substances in the blank plasma, and the calibration curves of lenvatinib and telmisartan were linear within the range of 0.2–1000 ng/mL and 0.1–500 ng/mL, respectively. The results indicate that lenvatinib decreased the systemic exposure of telmisartan. Potential drug interactions were observed between lenvatinib and telmisartan.

## 1. Introduction

Protein kinases are a group of enzymes concerned with the phosphorylation process that involves the migration of the phosphate group of ATP to proteins and plays an important role in regulating cellular signaling pathways. Tyrosine kinases are a subclass of kinases that regulate cell proliferation by phosphorylating the tyrosine portion of proteins. Overexpression or activation of tyrosine kinases is closely related to tumorigenesis and tumor progression; it is one of the characteristics of many types of tumors [1,2,3]. Thus, tyrosine kinase inhibitors (TKIs) play a vital role in tumor treatment in the clinical setting. Although TKIs are effective in diverse solid tumors, some adverse reactions might limit their use. Most patients have varying degrees of elevated blood pressure and may be accompanied by proteinuria when TKIs that target vascular endothelial growth factor receptors (VEGFR) to inhibit angiogenesis are applied [4,5,6]. Hypertension is associated with VEGFR-TKIs antitumor efficacy; studies have shown that patients who developed hypertension had a better response than those who did not [7]. The mechanism of VEGFR-TKIs induced hypertension is not clearly elucidated, but it is related to its antiangiogenic effect. The main reasons might be decreased nitric oxide production, decreased prostacyclin production, and increased endothelin 1 production, leading to vasoconstriction and thinning of the vascular bed, causing increased vascular resistance and increased blood pressure. On the other hand, decreased nitric oxide production leads to renal vasoconstriction, decreased renal blood flow, decreased filtration pressure, and water and sodium retention, causing increased blood pressure [5,8]. To date, there is limited medical evidence on drugs for the treatment of hypertension induced by VEGFR-TKIs due to the limited time available for development. However, renin-angiotensin system inhibitors including angiotensin-converting enzyme inhibitors (ACEI) and angiotensin receptor blockers (ARB) are probably the first-line treatment based on the mechanism of TKI-induced hypertension [5,8,9].

**Figure 1:**
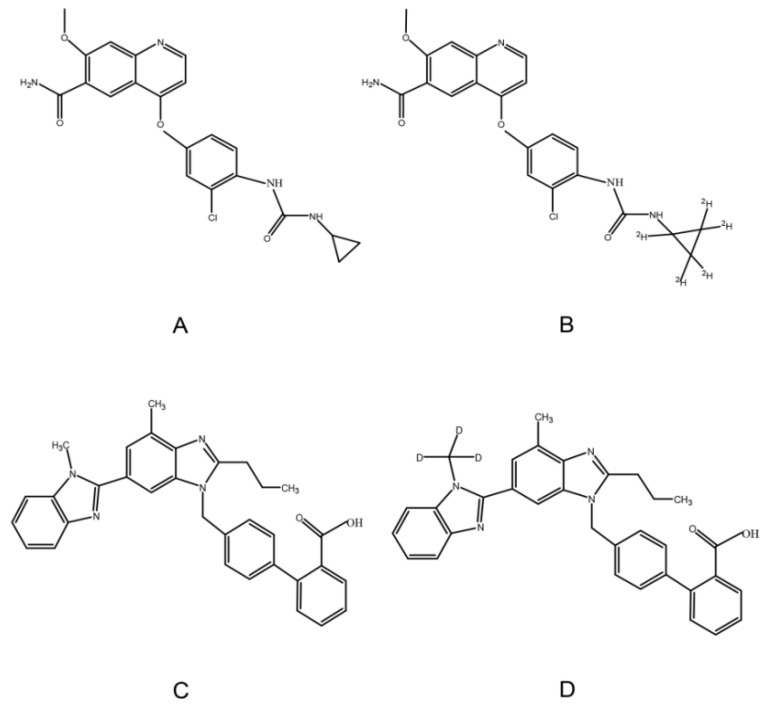
Chemical structure of LEN (**A**), ^2^H_5_-LEN (**B**), TEL (**C**), and TEL-d_3_ (**D**).

Lenvatinib (LEN) is an oral anti-cancer agent and multi-targeted TKI (Figure 1). It inhibits tumor angiogenesis by VEGFR 1–3. Moreover, it can also inhibit FGFR (fibroblast growth factor receptor) 1–4, PDGFR (platelet-derived growth factor receptor) α, rearranged during transfection, and c-Kit [10,11]. LEN was first approved for the treatment of invasive, locally advanced or metastatic, progressive, radiologically differentiated thyroid cancer by the U.S. Food and Drug Administration (FDA) in 2015 [12]. In 2018, a phase 3 clinical trial demonstrated that LEN was non-inferior to sorafenib in overall survival (primary endpoint), and it was recommended for the first-line treatment of unresectable advanced hepatocellular carcinoma [13]. In addition, LEN may be used in combination with everolimus for the treatment of advanced metastatic renal cell carcinoma [14,15]. A higher incidence of hypertension is associated with LEN than with other TKIs [4,16,17]. It is important to actively manage hypertension, and patients may tolerate the highest dose of treatment for the longest time to reap the maximum benefit from anticancer therapy.

Telmisartan (TEL), a highly selective ARB, can be used in the treatment of hypertension (Figure 1). Compared with other ARBs, TEL has higher lipophilicity and can cross the membrane more easily [18,19]. Moreover, TEL has a long half-life (24 h) that can sustain and significantly lower blood pressure, and it can also reduce cardiovascular risk and protect renal effect [20]. Therefore, LEN and TEL are likely to be used in combination in clinical settings due to the high incidence of hypertension induced by LEN and the unique pharmacological properties of TEL. In vitro studies have shown that TEL is the substrate and inhibitor of P-glycoprotein (P-gp), breast cancer resistance protein (BCRP), and multidrug resistance-associated protein 2 (MRP2) [21,22]; similarly, LEN is a substrate for P-gp, and BCRP [23,24]. Thus, there may be drug-drug interactions based on transporters when LEN is administered in combination with TEL. On the other hand, the plasma protein binding ratio of LEN and TEL are approximately 98% and 99%, respectively [18,25]; they can compete for the same binding site, resulting in significant changes in free drug concentration. Only free drugs can be distributed to tissues, metabolized, and cleared [26]. It is particularly important to evaluate the drug-drug interactions between LEN and TEL considering the above.

A few methods can be used to determine the blood concentration of LEN [27,28,29,30,31], but they have some limitations including a large amount of plasma being needed [27,28], a narrow range of calibration curves, and a long analysis time [29,30]. A validated ultra-performance liquid chromatography-tandem mass spectrometry (UPLC–MS/MS) method can be used to determine LEN, but it is not suitable for the quantification of TEL under these conditions [31]. Similarly, several methods are available for determining the blood concentration of TEL [32,33,34,35,36]. However, there are still some disadvantages such as consuming a lot of organic reagents, cumbersome pre-processing steps, longer sample pre-treatment time [32,33,34,35], and a narrow range of calibration curves [36]. The above methods have characteristics and limitations, but they are not suitable for the simultaneous quantification of LEN and TEL. To the best of our knowledge, no method has been reported for the simultaneous quantitation of LEN and TEL in plasma. Thus, we aimed to develop and validate a sensitive UPLC–MS/MS method for the simultaneous determination of LEN and TEL. Drug–drug interactions between the two drugs were evaluated by measuring the drug concentrations of LEN and TEL using a validated method.

## 2. Results and Discussion

### 2.1. UPLC-MS/MS Method Development

The previous method for TEL mostly used liquid–liquid extraction, which is time-consuming and tedious because it requires a large amount of organic reagents (2 mL or more) [32,34,35]. In this study, the plasma samples were pretreated by protein precipitation using acetonitrile. A simple plasma protein precipitation technique can be processed quickly, and it is suitable for high throughput sample determination. On the other hand, research showed that acetonitrile, a protein precipitant, had high precipitation efficiency and required a low dosage.

LEN belongs to the biopharmaceutics classification system (BCS) II or IV drugs with poor water solubility [37]; similarly, TEL is a poorly water-soluble drug that belongs to BCS II, and solubility is related to PH [38]. Therefore, the PH of the mobile phase affects the separation of TEL. The analytes can be well separated and responded when the mobile phase was ultrapure water containing 0.1% formic acid and 5 mM of ammonium acetate (A) and acetonitrile with 0.1% formic acid (B). Furthermore, the addition of formic acid resulted in a better response of the analytes, while the addition of ammonium acetate improved the peak shape of the analytes and reduced the matrix effect of TEL. For gradient elution to separate LEN and TEL, and to eliminate the carryover of TEL, a high percentage of phase B was used.

**Figure 2:**
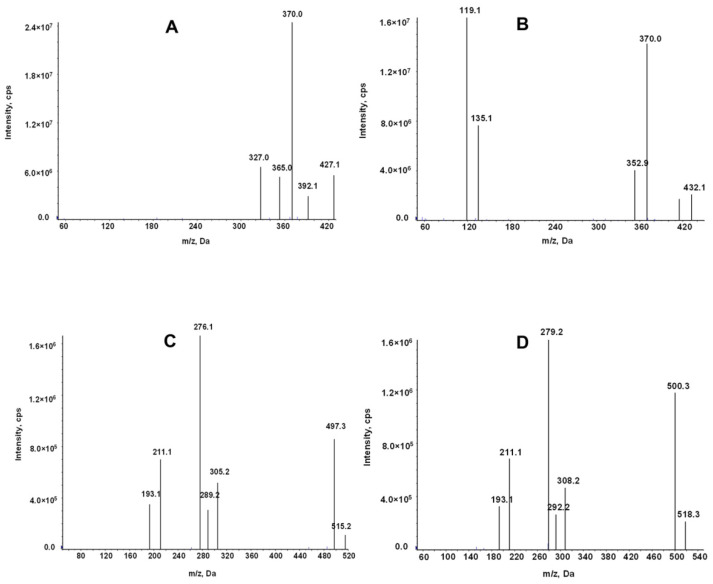
Product ion mass spectrum of LEN (**A**), ^2^H_5_-LEN (**B**), TEL (**C**), and TEL-d_3_(**D**).

The mass spectrometry for LEN and TEL was conducted in the positive ion mode and multiple reaction monitoring (MRM) modes. The use of stable isotopes as IS eliminates the ionization differences and matrix interference. The ion transitions monitored for quantification were *m*/*z* 427.1→370 for LEN, 432.1→370 for ^2^H_5_-LEN, 515.2→497.3 for TEL, and 518.3→279.2 for TEL-d_3_ (Figure 2).

### 2.2. UPLC-MS/MS Method Validation

#### 2.2.1. Selectivity

**Figure 3:**
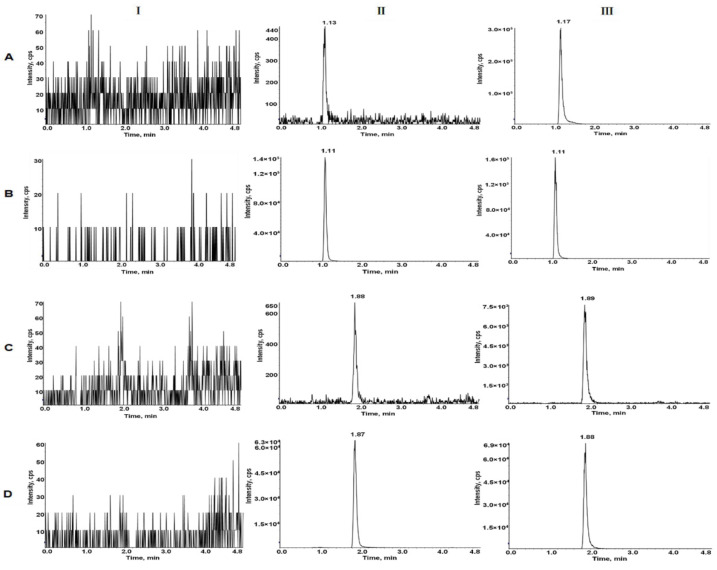
Typical MRM chromatograms of LEN (**A**), ^2^H_5_-LEN (**B**), TEL (**C**), and TEL-d_3_ (**D**). I, blank plasma; II, blank rat plasma spiked with the mixed working solution at LLOQ level and IS; III, rat plasma sample after oral administration of LEN and TEL.

Typical MRM chromatograms of LEN and TEL in blank plasma, blank plasma spiked lower limit of quantitation (LLOQ) and IS, and actual plasma of rats after the oral administration are shown in Figure 3. The retention times for LEN and TEL were 1.10 min and 1.88 min, respectively. No interfering peaks were detected at the retention times of the analytes and IS.

#### 2.2.2. Calibration Curve and LLOQ

The calibration curves of LEN and TEL were linear over the concentration range of 0.2–1000 ng/mL and 0.1–500 ng/mL, respectively. The typical calibration curves for LEN and TEL were:

Y = 0.0149 X + 0.000293 (r > 0.999)(1)

Y = 0.0636 X + 0.00276 (r > 0.999)(2)

The LLOQ values of LEN and TEL were 0.2 ng/mL and 0.1 ng/mL, respectively. LLOQ is the lowest concentration of the calibration curve, and the difference between the back-calculated concentration with the calibration curve and the nominal concentration should be less than 20%.

#### 2.2.3. Precision and Accuracy

Precision included the RSD of measurements taken on the same day (intra-batch) and those taken on three consecutive days (inter-batch). Accuracy included RE for measurements taken on the same day (intra-batch) and for measurements taken on three consecutive days (inter-batch). The results are shown in Table 1. The intra-batch and inter-batch precision (RSD) were within 1.72% to 7.09% and 3.61% to 9.58% for LEN, respectively, and 2.93% to 6.68% and 3.86% to 5.88% for TEL, respectively. All the results were within acceptable limits. The intra-batch and inter-batch accuracy (RE) were within 0.08% to 3.33% and −1.40% to 5.06% for LEN, respectively, and −1.63% to 6.45% and −1.44% to 5.94% for TEL, respectively. All the results were within acceptable limits.

#### 2.2.4. Matrix Effect and Extraction Recovery

The range of matrix effect for LEN and TEL was 100.3–106.79% and 105.77–109.29%, respectively (Table 2). The results of the matrix effect show that the substrate did not affect the quantification of LEN and TEL. The range of extraction recovery for LEN and TEL was 97.94–106.57% and 96.04–104.05%, respectively (Table 2). The results show that the extraction recovery of analytes at three different concentrations was consistent and reproducible.

#### 2.2.5. Stability

The data obtained from the LEN and TEL stability experiments are shown in Table 3. The results indicate that the plasma samples were stable when placed at room temperature for 8 h, at the autosampler for 24 h after processing, at −80 °C for 30 days, and freezing and thawing three times.

### 2.3. Pharmacokinetic Interaction of TEL with LEN

**Figure 4:**
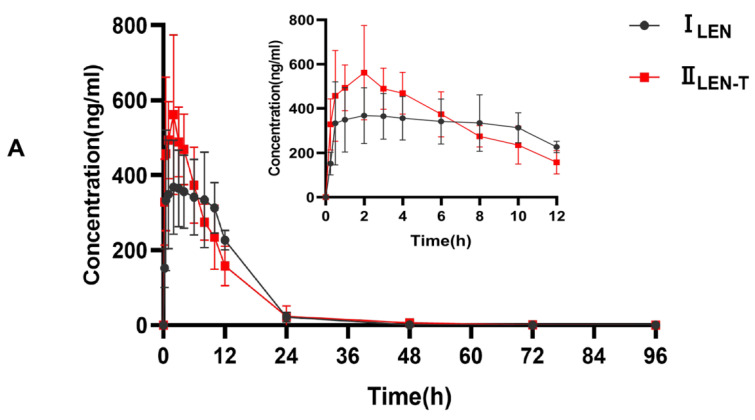

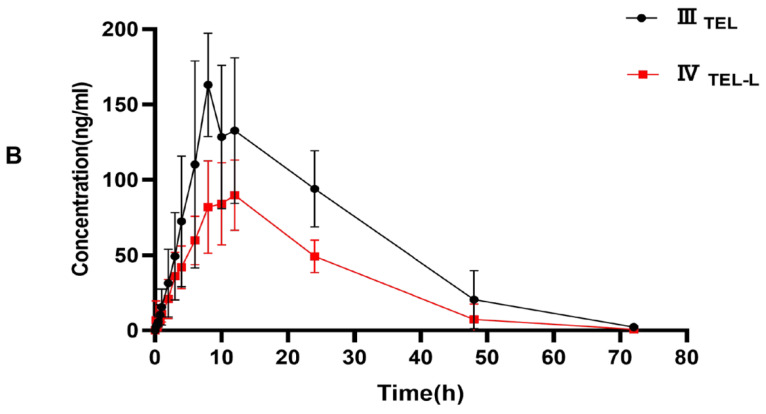
(**A**) The mean plasma concentration–time curves of LEN after oral administration, I_LEN_: 1.2 mg/kg LEN, II_LEN+T_: 1.2 mg/kg LEN combined with 4 mg/kg TEL; (**B**) the mean plasma concentration-time curves of TEL, III_TEL_: 4 mg/kg TEL, IV_TEL+L_: 4 mg/kg TEL combined with 1.2 mg/kg LEN after oral administration.

This fully validated method was successfully applied to LEN and TEL drug interaction studies in rat plasma. The doses of LEN and TEL were 1.2 mg/kg and 4 mg/kg, respectively, and they were chosen by converting the clinically recommended doses for patients to animal doses [39]. The mean plasma concentration–time curves of LEN when administered alone and in combination with TEL, as shown in Figure 4A, and the mean plasma concentration–time curves of TEL when administered alone and in combination with LEN are shown in Figure 4B. The main pharmacokinetic parameters of LEN and TEL are shown in Table 4. The results show that TEL had no significant effect on the pharmacokinetics of LEN. However, the main pharmacokinetic parameters including the maximum plasma concentration (C_max_), the area under the concentration-time curve (AUC), apparent volume of distribution (V_z_), and volume of plasma cleared per time unit (CL) of TEL were significantly changed after the simultaneous administration of LEN compared with the control group. The results showed that TEL exposure was significantly reduced when LEN was also given. The AUC_0–72h_ and AUC_0–∞_ of TEL were reduced by 44.9% and 41.3%, respectively, accompanied by a significant increase in CL (69%). The C_max_ of TEL was reduced by 42.2%, but V_z_ was significantly higher by 97.1% compared to the administration of TEL alone.

LEN and TEL are both substrates of P-gp and BCRP. In vitro studies have shown that TEL is an inhibitor of P-gp and BCRP. Previous studies have indicated that TEL may lead to an increase in digoxin and clopidogrel exposure through the inhibition of P-gp [40]. Another study demonstrated that TEL may increase exposure to rosuvastatin by inhibiting BCRP [41]. Thus, transporter-mediated drug-drug interactions may exist between LEN and TEL. Interestingly, the results of drug-drug interactions exceeded our expectations. The results of this study show that TEL has no effect on LEN exposure; however, the systemic exposure of TEL significantly decreased after 1.2 mg/kg LEN was also given. No study has reported on the induction of P-gp and BCRP by LEN, and the reason for the reduction in TEL exposure due to LEN is not known. We speculate that the possible reasons for this phenomenon are as follows: LEN and TEL compete for the same protein binding site, leading to an increase in the free drug concentration of TEL, in turn leading to accelerated TEL elimination or altering the tissue distribution of TEL, which is similar to another study [42]; another speculation is that LEN altered the tissue distribution of TEL by influencing the transporter, resulting in an abnormal pharmacokinetic profile of TEL. However, these are speculations on our part, and the free drug concentrations were not measured, which is a limitation of this paper. In the next step, we will measure the free drug concentrations and tissue distribution to explore the specific mechanisms.

## 3. Materials and Methods

### 3.1. Drugs and Reagents

LEN (purity 98%, Q75191201) was kindly provided by the Shijiazhuang Pharmaceutical Group. The internal standard (IS, ^2^H_5_-LEN, ZZS-20-624-A9) for LEN was acquired from Shanghai Zhen Zhun Biological Technology Co. Ltd. (Shanghai, China). TEL (Y20A7C13363, ≥98%) was supplied by Shanghai yuan ye Bio-Technology Co. Ltd. TEL-d_3_ was purchased from TLC Pharmaceutical Standards (Aurora, ON, Canada) and used as the IS for TEL. Dimethyl sulfoxide was acquired from Beijing Solarbio Science Technology Co. Ltd. (Beijing, China). HPLC-grade acetonitrile, ammonium acetate, and formic acid were supplied by Fisher Scientific (Pittsburgh, PA, USA).

### 3.2. Chromatographic Equipment and Conditions

An ultra-high-performance liquid chromatography (LC-30A, Shimadzu, Japan) equipped with a binary high-pressure pump (LC-30AD), column temperature chamber (CTO30A), and an autosampler (SIL30AC) was used. The composition of mobile phase was ultrapure water containing 0.1% formic acid and 5 mM of ammonium acetate (A), and acetonitrile with 0.1% formic acid (B). The mixture of drugs was separated by gradient elution with the following elution procedure: 0–2 min, 60% B; 2–3 min, 60–90% B; 3–4 min, 90% B; 4.0–4.1 min, 90–60% B; 4.1–5.1 min, 60% B. The column temperature chamber was set at 40 °C, and the injection volume was 6 µL. Chromatographic separation was performed using a column XSelect HSS T3 (2.1 mm × 100 mm, 2.5 μm, Waters) at a flow rate of 0.25 mL/min.

### 3.3. Mass Spectrometry Equipment and Conditions

An AB Sciex Triple Quad 5500 mass spectrometer equipped with an electrospray ionization source (ESI) was used for mass spectrometry. The analytes were detected in the positive ion and multiple reaction monitoring (MRM) mode. The monitored ion pairs were *m*/*z* 427.1→370 for LEN, 432.1→370 for ^2^H_5_-LEN, 515.2→497.3 for TEL, and 518.3→279.2 for TEL-d_3_ (Figure 2). The declustering potential and collision energy for LEN and TEL were 100 V and 45 V, respectively, and the other mass spectrum parameters were as follows: collision gas, 8 kPa; curtain gas, 20.0 psi; source temperature, 500 °C; ion spray voltage, 5500 V; ion source gas 1, 50.0 psi; ion source gas 2, 60.0 psi.

### 3.4. Preparation of Stock Solution and Working Solution

Dimethyl sulfoxide was used to prepare the stock solution with a concentration of 2 mg/mL for LEN and 1 mg/mL for ^2^H_5_-LEN, TEL, and TEL-d_3_. The mixture working solution for the upper limit of quantitation contained 20 μg/mL of LEN and 10 μg/mL TEL; this was obtained by diluting the stock solution with 50% acetonitrile–water. Then, a series of mixed working solutions was obtained by diluting with 50% acetonitrile–water. The mixed IS working solution contained 500 ng/mL of ^2^H_5_-LEN and 525 ng/mL of TEL-d_3_; it was also diluted with 50% acetonitrile–water.

### 3.5. Preparation of Calibration Standards and Quality Control (QC) Samples

The calibration standards were prepared by spiking 5 μL of the mixed working solution with 45 μL of blank rat plasma. The final concentrations of the calibration curves were 0.2, 1, 2, 10, 20, 100, 200, 400, and 1000 ng/mL for LEN, and 0.1, 0.5, 1, 5, 10, 50, 100, 200, and 500 ng/mL for TEL. The QC samples were processed in the same manner as the calibration standards with the final concentrations of 0.5, 150, and 800 ng/mL for LEN, and 0.25, 75, and 400 ng/mL for TEL.

### 3.6. Plasma Sample Preparation

Protein precipitation was used to prepare the plasma samples. Then, 50 µL of the plasma samples, 5 µL of mixed IS working solution, and 150 µL of acetonitrile were vortex-mixed using a LP Vortex Mixer (Thermo scientific) for 2.0 min. The mixture was centrifuged at 13,000× *g* for 10 min; 100 µL of supernatant and 100 µL of 50% acetonitrile-water were added to a 1.5 mL centrifuge tube and centrifuged again. The supernatant was transferred to a 96-well plate for injection analysis.

### 3.7. Method Validation

The method was validated according to the guidelines of the Bioanalytical Method Validation Guidance for Industry by the U.S. FDA and Chinese Pharmacopoeia (2020).

#### 3.7.1. Selectivity

The selectivity was demonstrated by comparing multiple sources of rat blank samples with blank plasma spiked with the mixed working solution at LLOQ and IS (*n* = 6). The real samples obtained from rats after gavage were also used to evaluate the selectivity. The peak area of blank plasma at the retention time should be less than 20% of the LLOQ and 5% of the IS.

#### 3.7.2. Calibration Curve and LLOQ

The calibration curve linear ranges of LEN and TEL were evaluated at 0.2–1000 ng/mL and 0.1–500 ng/mL, respectively. The calibration curve was calculated by plotting the peak area ratio of analyte to IS vs. the nominal concentration of analytes using a 1/x2 weighted linear least squares regression; the difference between the back-calculated concentration using the calibration curve and the nominal should be less than 15% except for LLOQ. The difference for LLOQ between the back-calculated concentration with the calibration curve and the nominal concentration should be less than 20%.

#### 3.7.3. Precision and Accuracy

QC samples at three concentrations and LLOQ level samples were used to evaluate the precision and accuracy. The precision was expressed by calculating the relative standard deviation (RSD) of six replicates of samples, and the accuracy was expressed by calculating the relative error (RE) of six replicates of samples. The RSD and RE of the low, medium, and high QC samples should be less than 15%, and the RSD and RE of LLOQ samples should be less than 20%.

#### 3.7.4. Matrix Effect and Extraction Recovery

Matrix effect was assessed by comparing the peak areas of analytes in the presence of plasma at three concentrations of QC samples (low, medium, and high) with the peak areas of the corresponding pure solution analytes (*n* = 6). The extraction recovery was evaluated by comparing the peak areas of the analytes pre-spiked in the blank plasma of QC samples at low, medium, and high concentrations and the peak areas of post-extracted blank plasma spiked samples.

#### 3.7.5. Stability

QC samples with six replicates of each concentration were used to assess the stability of the samples under different processing and storage conditions. The short-term stability of the LEN and TEL at room temperature for 8 h and that of the autosampler for 24 h was investigated. The long-term stability was carried out by freezing at −80 °C for 30 days, and the freeze–thaw stability was verified by repeatedly freezing and thawing three times.

### 3.8. Application to Pharmacokinetic and Drug-Drug Interaction Study in Rats

SPF (specific pathogen free)-grade male SD (Sprague-Dawley) rats (weight: 230 ± 10 g) were purchased from Beijing Weitong Lihua Experimental Animal Technology Co. Ltd. (Beijing, China). Before starting the experiment, the rats were given sufficient food and water and kept under suitable conditions for one week. Suitable conditions included suitable temperature (23–27 °C), suitable humidity (50 ± 10%), and 12-h light–12-h dark diurnal cycle. All animals were fasted for 12 h before the start of the experiment, but they were allowed to drink water freely. All the animal experimental protocols were conducted in accordance with the UK Animals (Scientific Procedures) Act 1986 and under the guideline approved by the Ethics Committee of Hebei General Hospital (Shijiazhuang, China; No. 2021131).

Twenty-four healthy rats were randomly and equally divided into four groups (six animals in each group). TEL and LEN were uniformly suspended in 0.5% sodium carboxymethyl cellulose (CMC-Na). Group 1 (I_LEN_), the LEN control group, was given 1.2 mg/kg of LEN by gavage; Group 2 (II_LEN+T_), the LEN combined with TEL group, was given 1.2 mg/kg of LEN and 4 mg/kg of TEL by gavage. Approximately 0.3 mL of blood was collected from the orbital venous plexus in a heparinized centrifuge tube from each rat at 0.25, 0.5, 1, 2, 3, 4, 6, 8, 10, 12, 24, 48, 72, and 96 h after administration. Similarly, Group 3 (III_TEL_), the TEL control group, was given 4 mg/kg of TEL by gavage; Group 4 (IV_TEL+L_), the TEL combined with LEN group, was given 4 mg/kg of TEL and 1.2 mg/kg of LEN by gavage. The blood samples were collected from each rat at 0.17, 0.33, 0.5, 0.75, 1, 2, 3, 4, 6, 8, 10, 12, 24, 48, and 72 h after administration. Blood samples were centrifuged at 3500 rpm for 10 min, and the plasma samples (supernatant) were harvested and stored at −80 °C.

### 3.9. Statistical Analysis

The pharmacokinetic parameters of TEL and LEN were calculated using DAS 2.1.1 Software (Mathematical Pharmacology Professional Committee of China, Shanghai, China) using noncompartmental analysis. The main pharmacokinetic parameters are presented as mean ± standard deviation and statistically analyzed using SPSS 25.0 software package (SPSS Inc., Chicago, IL, USA). A *p*-value of <0.05 was considered statistically significant.

## 4. Conclusions

A method for the simultaneous determination of LEN and TEL was developed and validated. The method uses a simple one-step protein precipitation process with a short analysis time, a wide range of calibration curves, and only 50 μL of plasma volume, suitable for pharmacokinetic and drug-drug interaction studies. The method was successfully applied to LEN and TEL drug-drug interaction studies. The pharmacokinetic results indicate that LEN can result in a significant reduction in systemic exposure to TEL, which is very meaningful and deserves further study. It can provide an important reference for the combined use of these two drugs in clinical practice.

## Figures and Tables

**Table 1:** Intra-batch and inter-batch precision and accuracy of LEN and TEL in rat plasma.

Analytes	Concentration (ng/mL)	Intra-Batch (*n* = 6)			Inter-Batch (*n* = 18)		
		Mean ± SD	RSD (%)	RE (%)	Mean ± SD	RSD (%)	RE (%)
LEN	0.2	0.20 ± 0.01	7.09	0.08	0.21 ± 0.02	9.58	5.06
	0.5	0.51 ± 0.02	3.29	2.47	0.50 ± 0.03	5.28	0.22
	150	155.00 ± 8.15	5.26	3.33	151.11 ± 7.61	5.03	0.74
	800	810.33 ± 13.94	1.72	1.29	788.83 ± 28.50	3.61	−1.40
TEL	0.1	0.11 ± 0.01	6.68	6.45	0.11 ± 0.01	5.88	5.94
	0.25	0.25 ± 0.01	2.93	1.27	0.25 ± 0.01	3.86	−0.31
	75	75.10 ± 2.77	3.69	0.13	76.27 ± 4.38	5.75	1.70
	400	393.50 ± 14.18	3.60	−1.63	394.22 ± 18.98	4.81	−1.44

**Table 2:** Matrix effect and extraction recovery of LEN and TEL in rat plasma (*n* = 6).

Analytes	Concentration (ng/mL)	Matrix Effect		Extraction Recovery	
		Mean ± SD (%)	RSD (%)	Mean ± SD (%)	RSD (%)
LEN	0.5	106.79 ± 6.54	6.12	106.57 ± 5.99	5.62
	150	100.30 ± 8.40	8.38	97.94 ± 2.80	2.86
	800	100.80 ± 8.03	7.97	99.43 ± 11.59	11.65
TEL	0.25	109.29 ± 5.09	4.66	104.05 ± 5.37	5.16
	75	107.16 ± 6.48	6.05	96.04 ± 4.73	4.93
	400	105.77 ± 6.37	6.02	101.99 ± 10.61	10.40

**Table 3:** Stability of LEN and TEL in rat plasma under various storage conditions (*n* = 6).

Analytes	Conditions	Concentration (ng/mL)	Mean ± SD (ng/mL)	Precision (RSD%)	Accuracy (RE%)
LEN	Autosampler for 24 h	0.5	0.48 ± 0.03	6.65	−3.30
		150	148.33 ± 6.06	4.08	−1.11
		800	835.67 ± 34.60	4.14	4.46
	Room temperature for 8 h	0.5	0.52 ± 0.03	5.82	4.57
		150	149.33 ± 11.15	7.46	−0.44
		800	751.50 ± 19.74	2.63	−6.06
	−80 °C for 30 days	0.5	0.53 ± 0.05	8.81	5.67
		150	154.00 ± 4.77	3.10	2.67
		800	808.00 ± 59.16	7.32	1.00
	Freeze–thaw stability for three times	0.5	0.48 ± 0.05	10.53	−3.17
		150	150.17 ± 14.85	9.89	0.11
		800	787.50 ± 40.13	5.10	−1.56
TEL	Autosampler for 24 h	0.25	0.24 ± 0.02	6.61	−4.33
		75	73.68 ± 1.59	2.16	−1.76
		400	398.50 ± 22.76	5.71	−0.38
	Room temperature for 8 h	0.25	0.24 ± 0.01	5.18	−3.53
		75	73.23 ± 5.40	7.37	−2.36
		400	369.67 ± 6.98	1.89	−7.58
	−80 °C for 30 days	0.25	0.25 ± 0.01	5.05	1.80
		75	74.73 ± 1.64	2.20	−0.36
		400	401.50 ± 27.46	6.84	0.38
	Freeze–thaw stability for three times	0.25	0.25 ± 0.01	3.21	−0.47
		75	72.35 ± 2.51	3.47	−3.53
		400	391.33 ± 25.73	6.57	−2.17

**Table 4:** Pharmacokinetic parameters of LEN and TEL in rat plasma after the oral administration of single dose and combined doses.

Parameters (Unit)	LEN (1.2 mg/kg)		TEL (4 mg/kg)	
	Alone	with TEL (4 mg/kg)	Alone	with LEN (1.2 mg/kg)
AUC_0–t_ (ug/L*h)	5665.51 ± 602.61	5859.99 ± 1350.86	4146.02 ± 1035.68	2284.10 ± 322.18 **
AUC_0–∞_ (ug/L*h)	5666.29 ± 602.95	5863.68 ± 1350.05	4180.86 ± 1035.24	2455.98 ± 544.34 **
C_max_ (ug/L)	497.83 ± 106.93	644.50 ± 210.71	171.33 ± 38.05	98.98 ± 19.30 **
T_max_ (h)	2.5 (0.85–8.5)	1.5 (0.88–3.25)	8 (7.5–12)	11 (7.5–12)
t_1/2z_ (h)	8.75 ± 2.55	9.32 ± 4.69	9.36 ± 2.09	12.43 ± 12.71
CL_z_ (L/h/kg)	0.21 ± 0.02	0.22 ± 0.05	1.00 ± 0.21	1.69 ± 0.35 **
V_z_ (L/kg)	2.69 ± 0.79	2.92 ± 1.60	13.49 ± 3.97	26.59 ± 19.50 *

* *p* < 0.05, ** *p* < 0.01, compared with the vehicle alone, indicating statistically significant difference. The main pharmacokinetic parameters are expressed as the mean ± standard deviation, and T_max_ (h) is expressed as the median (range).

## Data Availability

Not applicable.

## Sample Availability

Samples of the compounds are not available from the authors.
